# Pilot Study on the Relationship between Malnutrition and Grip Strength with Prognosis in Diabetic Foot

**DOI:** 10.3390/nu15173710

**Published:** 2023-08-24

**Authors:** Magali González-Colaço Harmand, Alicia Tejera Concepción, Francisco José Farráis Expósito, Jennifer Domínguez González, Yolanda Ramallo-Fariña

**Affiliations:** 1 Department of Internal Medicine-Geriatric Medicine, Hospital Universitario Nuestra Señora de Candelaria, 38010 Santa Cruz de Tenerife, Spain; atejcon@gobiernodecanarias.org; 2 Faculty of Health Sciences, Universidad Europea de Canarias, 38300 La Orotava, Spain; 3 Internal Medicine Department, Universidad de la Laguna, 38200 La Laguna, Spain; 4 Department of Vascular Surgery, Hospital Universitario Nuestra Señora de Candelaria, 38010 Santa Cruz de Tenerife, Spain; ffarexp@gobiernodecanarias.org; 5 Medicine Faculty, Universidad de la Laguna, 38200 La Laguna, Spain; jennifer.dg.009@gmail.com; 6 Fundación Canaria Instituto de Investigación Sanitaria de Canarias (FIISC), 35019 Las Palmas de Gran Canaria, Spain; y.ramallo@gmail.com; 7 Network for Research on Chronicity Primary Care and Health Promotion, 28029 Madrid, Spain

**Keywords:** diabetic foot, sarcopenia, malnutrition

## Abstract

Sarcopenia and malnutrition have been associated in the elderly population with a poor prognosis in wound healing and with other adverse events, such as institutionalization or functional impairment. However, it is not known how these factors influence the prognosis of diabetic foot in the elderly. To answer this question, a prospective observational study of 45 patients over 65 years of age admitted with diagnoses of diabetic foot in a tertiary hospital has been conducted. All patients were assessed at admission and at 3 months after returning home to determine quality of life, pain, mobility and healing, overall hospital stay in relation to the presence of malnutrition (measured by BMI, CIPA scale and analytical parameters at admission of serum proteins and albumin), and sarcopenia measured by grip force, among other geriatric syndromes. The results found a relationship between altered sarcopenia and more pain and poorer quality of life, and altered BMI was related to a lower cure rate and worse mobility at follow-up. This study seems to indicate that, in the elderly population with diabetic foot, malnutrition and sarcopenia should be managed at the same time as the treatment of the diabetic foot itself.

## 1. Introduction

Diabetic foot (DF) is one of the main complications in patients with diabetes, with an incidence of up to 15% in cases of type 2 diabetes mellitus (T2DM). This complication arises from complex pathophysiological mechanisms, which include the development of both neuropathy and peripheral arterial disease [1]. The consequences of this disease for patients and their environment are devastating, encompassing the need for amputations, a loss of quality of life and functionality, mortality, and associated healthcare costs, along with decreased productivity.

A meta-analysis conducted in 2017 on the global epidemiology of DF ulcers demonstrated that they typically occur in elderly men with T2DM [2]. The global diabetic foot ulcer prevalence is 3–4% [3], and the lifetime risk of foot ulcer is 19–34% [4].

Specifically, the amputation rate in Spain is around 3/1000 people with diabetes, being approximately twice as high in men [5]. Moreover, amputations in individuals older than 65 years have increased in recent years [6,7]. Therefore, patients with DF are typically older and, in these individuals, the development of DF more accurately predicts mortality than experiencing a stroke, coronary artery disease, or peripheral arterial disease [8]. Furthermore, the higher the age is, the higher the mortality rate associated with DF will be [9]. These factors are coupled with a lower possibility of wound healing and an increased risk of amputation [10,11].

However, the literature on older individuals with DF is limited, and little is known about either the characteristics or the comorbidities that favor its development in this age group [12].

Most studies in the literature on DF include patients younger than 55 years [13,14,15]. These studies have proposed risk factors for the development of this condition, such as the presence of a previous ulcer, loss of sensation, foot deformities, and circulatory impairment. The few studies conducted in older patients have focused on describing the clinical features of diabetic neuropathy [14]. In these studies, the factors associated with increased mortality were the presence of peripheral arterial disease, previous amputations, poor glycemic control, and the development of nephropathy. However, geriatric syndromes have not been studied as prognostic determinants [16]. Considering that the presence of geriatric syndromes influences vital prognosis, functional status, and quality of life in the older population [17,18,19], there is a need to elucidate how these syndromes also impact the progression of DF in this population.

The main aim of this study was to describe the clinical and geriatric characteristics of elderly patients admitted with a diagnosis of DF. A second aim was to assess their short-term prognosis over a 3-month follow-up.

## 2. Materials and Methods

We conducted an observational, descriptive, longitudinal, and prospective study in patients aged 65 years or older who were admitted to the Vascular Surgery Unit at the University Hospital Nuestra Señora de Candelaria (a tertiary hospital in Tenerife, Spain) with a diagnosis of DF between 1 July 2022 and 31 December 2022. Nondiabetic patients with ischemic ulcers were excluded. Patients with an ominous vital prognosis or terminal illness were not excluded in order to reflect real-life practice.

### 2.1. Clinical Assessment

The following variables were recorded on admission:

Sociodemographic variables: (a) age; (b) sex; (c) time since diagnosis of DM; (d) living arrangement (living alone or not); (e) social assessment measured using the Gijón social-familial evaluation scale (SFES) scale [20], which scores up to 24 points. Scores between 10 and 14 points indicate social risk, and scores higher than 15 indicate the presence of social problems.

Clinical variables: (a) cardiovascular risk factors; (b) the presence of comorbidities associated with DM; (c) macro- or microvascular complications of DM; (d) the Charlson comorbidity index was used to assess comorbidity, with a maximum severity score of 37 points [21]; (e) stage of the DF ulcer measured by the Wagner–Merrit classification system, which classifies the type of DF lesion into 5 grades: 0 = foot at risk, 1 = the presence of superficial ulcers, 2 = deep ulcers, 3 = deep ulcers with abscess or osteomyelitis, 4 = limited gangrene, and 5 = extensive gangrene [22]; (f) degree of infection measured by the Van Ackers/Peters system, which assesses the characteristics of the wound to determine the presence of infection, categorizing the type of lesion from 1 to 5 and the disease present in the foot from A to E [23]; (g) type of wound care applied.

Geriatric syndromes: (a) falls syndrome (defined as at least 3 falls in the previous 6 months); (b) cognitive impairment assessed using the Pfeiffer scale, a 10-item questionnaire in which errors are counted. The cutoff point is 3 or more errors for individuals who can read and write, and 4 or more for those who cannot [24]; (c) sarcopenia, measured by hand-grip strength dynamometry, taking into account established reference values in healthy adult Spanish population-based cohorts [25]; (d) the presence of urinary or fecal incontinence; (e) polypharmacy, defined as the use of at least 5 medications; (f) nutritional status, with albumin deficiency defined as levels below 3.5 g/dL and total protein deficiency as levels below 6 g/dL upon admission; (g) in-hospital nutritional screening performed using the CIPA scale [26]; (h) MUST criteria for malnutrition [27]; (i) body mass index (BMI), with underweight defined as a BMI equal to or below 22 in individuals older than 65 years, normal weight as 22.1–24.9, overweight as 25–29.9, class I obesity as 30–34.9, class II obesity as 35–39.9, and class III obesity as >40 [28].

Functional assessment: (a) the Barthel Index was used to measure independence for basic activities of daily living. This scale evaluates 10 parameters (feeding, bathing, grooming, dressing, bowels, bladder, toilet use, transfers (bed to chair and back), mobility (walking on level surface or propelling a wheelchair if unable to walk) and ascending and descending stairs. The scores are summed, and the degree of dependence is determined as follows: 100 = independence, ≤60 = mild dependence, 40–55 = moderate dependence, 20–35 = severe dependence, and <20 = total dependence [29]; (b) instrumental activities were measured using the Lawton–Brody scale. This scale assesses 8 items (ability to use the telephone, go shopping, prepare meals, do housekeeping, do laundry, use transportation, manage medication, and handle finances), with each item scoring 0 or 1. For men, the items related to meal preparation, housekeeping, and laundry are not scored, resulting in a maximum score of 8 for women and 5 for men. Independence is indicated by a score of 8 for women and 6 for men, mild dependence by scores of 6–7 for women and 4 for men, moderate dependence by scores of 4–5 for women and 2–3 for men, severe dependence by scores of 2–3 for women and 1 for men, and total dependence by scores of 0–1 for women and 0 for men [30].

Measurements of the following variables at admission and at a 3-month follow-up, which was conducted via telephone, were compared:

- Pain: assessed using a 0–10 visual analog scale (VAS), with 10 indicating the most severe pain.
- Quality of life: at admission, quality of life was measured using the DFS-SF scale (Diabetic Foot Ulcer Scale Short Spanish Validated Form) [31]. The DFS-SF is a disease-specific questionnaire that assesses the impact of DF ulcers on quality of life. The scale consists of 29 items (scored on a scale of 1–5) based on the following 5 subscales: leisure, physical health, dependence/daily living, negative emotions, and bothered by ulcer care. The score is calculated based on a minimum value of 29 (better quality of life) and a maximum of 145 (worse quality of life). The version used is a shortened version of the original, which contains 58 items and 11 domains.

Quality of life at the end of the follow-up was assessed using a 0–10 numerical rating scale, where 0 represents the worst possible quality of life and 10 represents the best quality of life. Because the follow-up was conducted via telephone, we were unable to repeat the assessments using the DFS-SF quality of life scale. Thus, using a minimum of 29 points and a maximum of 145 points on the DFS-SF scale, a correlation was made between the points on the DFS-SF scale and the categorization on the numerical rating scale (EVA scale), as follows: 29 = 0; 41 = 1; 52 = 2; 64 = 3; 75 = 4; 87 = 5; 99 = 6; 110 = 7; 122 = 8; 133 = 9; 145 = 10.

- Mobility: measured by the ability to walk and transfer, this determines whether the patient was fully independent, required assistance to walk, was confined to bed and chair, or was completely bedridden.
- Length of hospital stay was also recorded (measured in days from admission to discharge), as well as whether the patient died during the follow-up period and if there were any readmissions.

Statistical analyses were performed using SPSS software (version 21; SPSS, Chicago, IL, USA). The main characteristics of the sample and the study variables are expressed as frequencies and percentages for categorical variables and mean ± standard deviation for continuous variables. To perform the bivariate analysis, we used Student’s *t* test for continuous variables to compare the means of dependent samples. To test the association between categorical variables, we used the chi-square and Fisher exact tests. To assess the association between two continuous variables, we calculated correlations. Statistical significance was set at *p* ≤ 0.05 and marginally significance was accepted for *p* < 0.1.

### 2.2. Ethical Considerations

This work has been carried out in accordance with the Code of Ethics of the World Medical Association (Declaration of Helsinki) and the laws and regulations in force in Europe and Spain. The information sheet was delivered to the participating subjects and/or relatives responsible, explaining the objectives and procedures of the study, after which we requested that they sign the informed consent form, making and offering a model for the patients with adequate decision-making capacity and another for relatives in necessary cases.

The present study was approved by the Ethics Committee of the University Hospital of Canary Islands (Santa Cruz de Tenerife, Spain) in its session of 22 October 2022.

## 3. Results

### 3.1. Baseline Characteristics

We included 45 patients diagnosed with DM2 and DF. They were aged 65–93 years and had a mean age of 76.82 ± 8.08 years. The sample was predominantly male (71%). The sociodemographic and functional characteristics of the sample are shown in Table 1.

In most patients (53.3%), the time since diagnosis of DM was more than 10 years. Hypertension was present in 91.1% of the cohort, and 11.1% were active smokers. Among the complications associated with diabetes, 68.9% of patients had ischemic heart disease, 42.2% had renal disease, 24.4% were on dialysis, 31.1% had retinopathy, and 28.9% had chronic anemia. Twenty-nine patients (64.4%) had previously experienced DF ulcers. The mean Charlson index was 8.96 ± 2.71, indicating a high level of comorbidity. The mean score on the Gijón scale was 10.33 ± 2.95, with 37.8% of patients at social risk and 11.1% facing social problems.

There prevalence of DF ulcers was high, as determined by the Wagner–Merrit scale and the Van Ackers/Peters system: 44 patients (97.8%) had a Wagner–Merrit grade IV ulcer, with stage 4E in the Van Ackers/Peters system. These data indicated that the patients had limited gangrene in a specific area of the foot, along with periostitis and/or direct bone contact without visible defects on X-ray, and also that the foot exhibited neuroischemic characteristics.

### 3.2. Geriatric Syndromes and Functional Assessment (Table 2)

Polypharmacy was present in 97.8% of patients. Urinary incontinence was present in 44.4% and fecal incontinence in 11.1%. The mean number of errors on the Pfeiffer test was 2.56, indicating very subtle cognitive impairment. The mean Barthel score (60.67) indicated mild dependence for the activities of daily living, while the mean score on the Lawton–Brody scale (3.87) indicated moderate dependence for the instrumental activities of daily living. None of the patients experienced fall syndrome in the 6 months prior to admission. Malnutrition was found in 68.9% of patients, as assessed based on albumin levels below 3.5 g/dL, and 66.7% met the criteria for malnutrition when total protein levels below 6 g/dL were considered. The CIPA tool yielded positive results in 28.9% of the patients. The correlation of abnormal albumin and total protein values with BMI revealed that, of the 12 patients with decreased values in both parameters, only 1 (8.4%) was in the normal weight range, while 91.6% were overweight, of 7 (58.4%) were overweight, 2 (16.7%) had class 1 obesity, and 2 (16.7%) had class 2 obesity. On the MUST scale, all patients were at a low risk of malnutrition.

**Table 2:** Characteristics of geriatric syndrome and functional assessment on admission.

	n	Mean ± SD	%
Geriatric syndrome			
Cognitive impairment (Pfeiffer)	45	2.56 ± 2.33	
Sarcopenia	12		26.7
Mobility			
Independent	9		20
Needs assistance	24		53.3
Confined to bed + chair	12		26.7
Urinary incontinence	20		44.4
Fecal incontinence	5		11.1
Polypharmacy	44		97.8
Altered albumin levels	31		68.9
Altered total protein levels	30		66.7
Positive CIPA	13		28.9
BMI	45	30.29 ± 5.88	
Underweight	3		6.7
Normal weigh	8		17.8
Overweight	13		28.9
Obesity I	13		28.9
Obesity II	4		8.9
Obesity III	4		8.9
Functional assessment			
Basic activities (Barthel)	45	60.67 ± 27.99	
Instrumental activities (Lawton–Brody)	45	3.87 ± 2.34	

BMI = body mass index; CIPA = control of food intake, protein, and anthropometry.

The sarcopenia assessment showed that 26.7% of patients obtained a score above the established threshold considered optimal for their age and sex. Among these patients, all were octogenarians (3 men and 9 women). No relationship was found between BMI and the results of dynamometry, as patients with sarcopenia were distributed across all BMI groups (underweight, normal weight, overweight, class I obesity, and class III obesity).

### 3.3. Three-Month Follow-Up Results

On admission, the mean pain score was 6.74 ± 1.67, which significantly decreased to 3.67 ± 2.99 at discharge (*p* < 0.001). Quality of life showed little change between admission and discharge (*p* = 0.704).

Mobility significantly worsened during the study period. There were 9 (20%) independent patients upon admission and only 1 (2.6%) at discharge. The percentage of patients who were confined to a bed and chair increased from 26.7% on admission to 53.8% at discharge (*p* < 0.001).

The mean length of stay was 55.67 ± 36.48 days (median 47.5 days).

Complete ulcer healing was achieved in 14 (31.11%) of the patients via the use of the treatment administered during follow-up.

Overall, 6 patients died (13.3%), with 3 deaths occurring during the hospital stay and the remaining 3 outside the hospital. In total, 7 patients (18%) were readmitted, with 5 of them experiencing a single readmission and 2 being readmitted multiple times. Due to the low incidence of these events, they could not be correlated with geriatric syndromes.

### 3.4. Association between Variables and Patient Outcomes at Follow-Up

**Figure 1:**
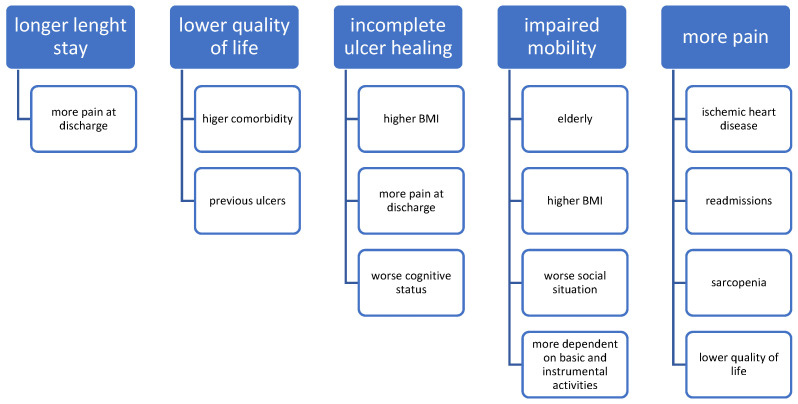
Variables determining the outcomes.

In our sample (Figure 1), excluding nonsurvivors, the length of hospital stay was negatively correlated with pain intensity at discharge (r = −0.37; *p* = 0.036). The mean hospital stay was marginally more significant in patients with diabetic retinopathy (*p* = 0.051) than in those without retinopathy (72 ± 43.30 vs. 48.32 ± 31.09, respectively).

Patients with complete ulcer healing had a lower mean BMI (26.89 ± 5.12 vs. 31.41 ± 5.19; *p* = 0.011), less pain at discharge (1.85 ± 2.38 vs. 4.58 ± 2.87; *p* = 0.005), better cognitive status (1.57 ± 1.51 vs. 3 ± 2.56; *p* = 0.030), and marginally higher quality of life at admission (6.38 ± 2.79 vs. 4.88 ± 2.20; *p* = 0.074). There was also a marginal association between complete healing and the absence of anemia (57.1%; *p* = 0.064).

Impaired mobility (bed–chair or bedridden vs. independent or needing assistance) was associated with older age (78.26 ± 7.86 vs. 71.88 ± 6.42; *p* = 0.011), higher BMI (31.83 ± 5.26 vs. 27.63 ± 5; *p* = 0.017), worse social assessment (11.13 ± 3.21 vs. 9 ± 2.42; *p* = 0.031) and less independence on admission, both for basic and instrumental activities of daily living: Barthel (47.17 ± 29.30 vs. 82.19 ± 14.72; *p* < 0.001) and Lawton–Brody (3 ± 2.28 vs. 5.13 ± 2.06; *p* = 0.005).

Pain was associated with the presence of ischemic heart disease (4.27 ± 3.24 vs. 2.46 ± 1.98; *p* = 0.039), readmissions (6.29 ± 3.50 vs. 3.09 ± 2.58; *p* = 0.008), and with the presence of sarcopenia (5.89 ± 2.47 vs. 3 ± 2.83; *p* = 0.009). Pain was also correlated with lower quality of life (r = −0.55; *p* < 0.001).

Lower quality of life was significantly correlated with a higher Charlson index (r = 0.31; *p* = 0.042) and the presence of previous ulcers prior to the development of the ulcer leading to the admission (5.52 ± 1.63 vs. 4.38 ± 2.0; *p* = 0.029). There was a marginal correlation between age (r = 0.26; *p* = 0.086) and Barthel index (r = −0.26; *p* = 0.078).

## 4. Discussion

Despite the small sample size (45 patients), this study provided relevant data on DF in individuals older than 65 years. First, our sample produced data comparable to those extracted from the general population, with DF being more common in older men and those with longstanding DM. In our sample, functional capacities were related to quality of life, as described in the literature [17]. Moreover, to ensure that the sample was as representative as possible of routine clinical practice, we did not exclude patients with a poor prognosis, as previously mentioned in the methodology section.

However, other data do not concur with previous findings: unlike previous studies [30] reporting low BMI in patients, our sample showed a high prevalence of overweight or obesity. The low smoking rate found in our study is also inconsistent with previous data, despite a high prevalence of hypertension and ischemic heart disease, with the mean comorbidity index indicating a low estimated 10-year survival rate. However, because of the advanced age of our patients, this datum may be biased due to both age itself and the intensification of morbidity in the end stages of life.

It is remarkable that our patients have a higher incidence of geriatric syndromes than found in similar published studies, not only in the community but in hospital settings too [32]. Polypharmacy, for example, has a reported incidence of 23% in the literature, and moderate dependence barely reaches 13% [33]. Considering studies that focus on diabetic patients, the association between diabetes and geriatric syndromes leads to a vicious circle with a background of neurovascular complications [34]. In this sense, our results agree with other publications that find malnutrition to be highly prevalent in diabetic patients (a third of diabetic patients admitted at Spanish hospitals were malnourished) [35], particularly in diabetic foot patients. A substantial number of patients were at risk of malnutrition (49–70%) or were malnourished (15–62%) [36]. Regarding other geriatric syndromes, the incidence of urinary incontinence in diabetic patients is 50.3%, which is consistent with the literature [37]. However, there is no available literature that shows the prevalence of geriatric syndromes different to the ones we have mentioned above or diabetic foot in older adults. In this context, our study is novel and provides data that support the importance of geriatric care in these patients.

We have found two particularly relevant aspects that act as social determinants of health-related, physical and cognitive impairments. Firstly, there is a group of patients in our study that, despite not being that old, were at a higher risk of social isolation and lack of social support, possibly because they had a physical dysfunction and disability before hospital admission. These patients also saw an even greater functional decline at hospital discharge, with few changes in their quality of life (which, as we know, is determined by function in the elderly). Adequate geriatric assessment is required in order to provide planned hospital and community resources, including ulcer care adapted to the individual needs.

Secondly, 44% of the patients in our study had cognitive impairments (3 or more mistakes as measured by the Pfeiffer test). The association between diabetes and cognitive impairment is well known [38,39], although its relationship with diabetic foot has been less explored. A review of the current literature focusing on younger patients [40], in whom a lower mortality is linked with a higher cognitive performance. There may be common pathophysiological pathways for both DM complications and cognitive impairment that contribute to increased mortality, an issue which should be explored.

We would like to highlight the importance of diagnosing malnutrition (low albumin and total proteins in blood), in overweight or obese, sarcopenic patients. Nutritional screening tools usually used in hospital levels, such as MUST (malnutrition universal screening tool) or CIPA (mixed nutritional screening method), fail to detect malnutrition in obese patients due to their high percentage of false negative results. In our study, the MUST screening determined that our patients were all at low risk, and the CIPA only detected 29% of malnourished patients. Malnourishment in obese patients often seems to be underestimated. Therefore, we should combine various methods to correctly diagnose malnourished, obese, and sarcopenic patients with diabetic foot.

Diabetes is associated with osteoporosis and a 32% increase in relative risk of total fractures compared with patients without diabetes [41]. In our sample, despite the high prevalence of diabetic retinopathy (31%), sarcopenia (26.7%) and poor mobility, we did not register falls or fractures throughout the follow-up period.

In our sample, geriatric syndromes played significant roles in the prognostic assessment of DF. Notable among these syndromes were sarcopenia and malnutrition. Previous data [42,43] suggest that patients with DF and sarcopenia have higher amputation and mortality [44] rates, although the evidence is limited, and no relationship has been found with pain. In contrast, a relationship between sarcopenia and pressure ulcers has been described previously, but not with other types of ulcers such as chronic venous ulcers [45,46].

In this study, nutritional status was a prognostic factor related to different key outcomes (mobility and ulcer healing) and easily measured using BMI. Our results highlight that malnutrition due to obesity is particularly harmful in these patients, likely because it implies poor glycemic control and inadequate adherence to dietary plans, which are determining factors in the prognosis of DF. Additionally, given the above-mentioned data on sarcopenia in these patients, they may have had sarcopenic obesity, a diagnosis of particular importance [47,48] due to its prognostic implications in older patients.

To our knowledge, this is the first study that has found this association and our findings should help to generate a research hypothesis that can be further investigated in order to improve the nutritional status in patients with diabetic foot.

Although many studies have shown the efficacy of nutritional supplementation in wound healing [49,50], there is no evidence of its impact in diabetic foot patients. In our sample, for example, despite detecting malnourished patients, only 4 were treated using dietary supplements. This demonstrated that there are still opportunities for improvement in the management of older adults with diabetic foot, especially when taking into account that malnutrition will not only affect the healing process itself, but the entire general state of the patient.

Malnutrition in the elderly, whether diabetic or not, is a major problem related to frailty [51], delirium [52], decreased immunocompetence, muscle wastage, hypothermia, osteoporosis, mood changes, cognitive impairment, lowered quality of life, and premature mortality regardless of the specific cause of death [53,54].

Of note, pain decreased significantly in our sample (from 6.87 to 3.67 points out of 10 points), but this improvement was not accompanied by a proportional increase in quality of life, which remained relatively stable at around 6 out of 10 points. The reason for this paradoxical finding may be that quality of life in older adults is multidimensional and is not determined by a single factor. While pain at discharge does play a role in quality of life, our sample clearly shows that it is influenced by factors such as sarcopenia and comorbidity.

Frailty and sarcopenia should be categorized as a third category of complications, in addition to the traditional microvascular and macrovascular complications that lead to disability in older adults with diabetes [55]. In our study, the rate of sarcopenia is similar to that found in other studies with diabetic patients, with a prevalence up to 29% [56]. Cheng et al. reported that the percentage of sarcopenia in diabetic foot was more than double that of those without diabetic foot disease. Sarcopenia was independently associated with this condition [57]. Furthermore, in the diabetic foot disease group, patients with sarcopenia exhibited more foot ulcers, higher Wagner grade, and greater percentage of amputation compared with patients without sarcopenia [57]. Therefore, managing these factors may be important in optimizing quality of life in older patients with DF, as demonstrated [58,59] in the general geriatric population.

The current literature suggests that the management of DF in older adults could be optimized by adopting a multidisciplinary approach [60]. In recent years, efforts have been made to establish a relationship between nutritional status and prognosis, with nutritional status being the only geriatric syndrome taken into account [61,62]. However, the results on this issue remain uncertain. Based on our findings, despite the main limitation of a small sample size, we suggest that a comprehensive geriatric assessment, along with an appropriate evaluation and care plan targeting the improvement of geriatric syndromes, could be beneficial in older patients with DF.

## 5. Conclusions

Diabetic foot in the older adult population is associated with higher comorbidity (such as hypertension, ischemic heart disease or kidney disease), and geriatric syndromes such as polypharmacy, physical dysfunction and disability, urinary incontinence or immobility. Malnutrition and sarcopenia may be particularly relevant factors implicated in the prognosis of diabetic foot patients. Targeted treatment should probably improve healing, physical dysfunction and disability, as well as pain during follow-up. We can therefore say that older adults with DF require an assessment that accounts for geriatric syndromes as they are related to short-term prognosis in this age group. Optimizing sarcopenia and malnutrition, as well as managing pain effectively, could help to improve quality of life in this population. Further studies are needed to explore these aspects in more detail.

## Figures and Tables

**Table 1:** Sociodemographic and clinical characteristics of the sample on admission.

	n	Mean ± SD	%
Age (years)	45	76.82 ± 8.08	
Female sex	13		28.9
Living alone	10		22.2
Time since diagnosis	45		
<5 years	10		22.2
5–10 years	11		24.4
>10 years	24		53.3
Social assessment (Gijón)	45	10.33 ± 2.95	
Normal	23		51.1
At social risk	17		37.8
Social problems	5		11.1
Clinical problems			
Hypertension	41		91.1
Anemia	13		28.9
Active smoking	5		11.1
Prior ulcers	29		64.4
Ischemic heart disease	31		68.9
Diabetic neuropathy	44		97.8
Diabetic kidney disease	19		42.2
Diabetic retinopathy	14		31.1
Dialysis	11		24.4
Charlson index	45	8.96 ± 2.71	
Wagner–Merrit			
Superficial ulcers	1		2.2
Limited gangrene	44		97.8
Van Ackers/Peters system			
2A	1		2.2
4E	44		97.8
Ulcer treatment			
Betadine	41		91.1
Betadine + Hydration	1		2.2
Negative pressure wound dressing (PICO)	1		2.2
Prontosan + Purilon	2		4.4

SD: standard deviation.

## Data Availability

Public data are not available.
